# The clinical value and safety of ECG-gated dipyridamole myocardial perfusion imaging in patients with aortic stenosis

**DOI:** 10.1038/s41598-019-48901-y

**Published:** 2019-08-27

**Authors:** Fang-Shin Liu, Shan-Ying Wang, Yu-Chien Shiau, Yen-Wen Wu

**Affiliations:** 1 Department of Nuclear Medicine, Far Eastern Memorial Hospital, New Taipei City, Taiwan; 2 Division of Cardiology, Cardiovascular Medical Center, Far Eastern Memorial Hospital, New Taipei City, Taiwan; 3 National Yang-Ming University School of Medicine, Taipei, Taiwan; 4 Department of Nuclear Medicine, National Taiwan University Hospital and National Taiwan University College of Medicine, Taipei City, Taiwan

**Keywords:** Valvular disease, Radionuclide imaging

## Abstract

The role of vasodilator myocardial perfusion imaging (MPI) for aortic stenosis (AS) is controversial due to safety and accuracy concerns. In addition, its utility after aortic valve (AV) interventions remains unclear. Patients with AS who underwent thallium-201-gated dipyridamole MPI using a cadmium-zinc-telluride camera were retrospectively reviewed and divided into three groups: mild AS, moderate-to-severe AS, and prior AV interventions. Patients with coronary artery disease with ≥50% stenosis, severe arrhythmia, left ventricular ejection fraction (LVEF) <40%, left bundle branch block or no follow-up were excluded. Relationships between the severity of AS, clinical characteristics, hemodynamic response, serious adverse events (SAE) and MPI parameters were analyzed. None of the 47 patients had SAE, including significant hypotension or LVEF reduction. The moderate-to-severe AS group had higher summed stress scores (SSSs) and depressed LVEF than the mild AS group, however there were no differences after AV interventions. SSS was positively correlated with AV mean pressure gradient, post-stress lung-heart ratio (LHRs), and post-stress end-diastolic volume (EDVs) (*P* < 0.05). In multivariate analysis, LHRs and EDVs were independent contributors to SSS. Dipyridamole-induced ischemia and LV dysfunction is common, and dipyridamole stress could be a safe diagnostic tool in evaluation and follow-up in patients with AS.

## Introduction

Aortic valve stenosis (AS) is the most common valvular disorder. Clinical factors such as older age, male sex, smoking, hypertension, diabetes and hypercholesterolemia are important in the progression of AS^1,2^.

AS can result in microcirculatory dysfunction, defined as abnormalities in myocardial blood flow (MBF) despite the presence of normal left ventricular (LV) function and normal coronary arteries^3,4^. LV remodeling is attributed to several sequelae: (a) hemodynamic factors including increased LV pressure, reduced coronary perfusion pressure and extravascular compressive forces^4,5^; and (b) pathological changes including myocardial fibrosis and decreased density of vessels in myocardial tissue^6^.

The natural history of AS begins with a prolonged asymptomatic period. In general, mortality is not significantly increased in patients with asymptomatic AS or in those who receive aortic valve (AV) interventions, whereas the mortality is high after the development of symptoms. The management guidelines for patients with severe AS from the American College of Cardiology/American Heart Association Guidelines recommend that patients with equivocal symptoms should receive modified exercise tests^5^. Unfortunately, because of similar risk factors, concomitant coronary artery disease (CAD) in patients with AS is common, and similar symptoms may impede the proper management in this population^2,7^.

There is increasing consensus on the management strategy for patients with coexisting transcatheter aortic valve implantation (TAVI) and percutaneous coronary intervention (PCI)^8^, and single-photon emission computed tomography (SPECT) myocardial perfusion imaging (MPI), a non-invasive examination of last resort, may play an important role in this strategy, especially for those with high operative risk^9^. Nevertheless, the role of vasodilator MPI in patients with significant AS is still controversial due to safety concerns and uncertainty over the clinical accuracy. Vasodilator stress decreases systemic blood pressure, which may be unsafe in patients with AS. Given the potential risks, previous guidelines have discouraged stress testing in patients with severe and symptomatic AS, and few studies have investigated the safety of vasodilators stress in these patients^10–14^. Several studies on the presentation of AS with regards to MBF and subsequent myocardial flow reserve (MFR) derived from dynamic MPI have been published in recent years, however no definite characteristics have been found on conventional MPI imaging^4,6,15–18^. In addition, its utility in detecting myocardial ischemia in patients after aortic valve (AV) interventions has not been established^15^. The purpose of this study was to evaluate the efficacy and safety of dipyridamole stress SPECT MPI in patients with AS and after AV interventions.

## Results

### Patients characteristics

Forty-seven patients with AS were included in this study (Table 1). The mean age of the patients was 75.3 ± 10.2 years, and 34% (n = 16) were male. Twenty-seven patients had mild AS, 15 had moderate-to-severe AS, and five had previously received aortic valve replacement (AVR) or TAVI. All patients received clinical follow-up at least 6 months after SPECT MPI, and none had major cardiovascular events.

**Table 1:** Clinical characteristics of enrolled patients in three groups.

	Mild AS (n = 27)	Moderate-to-severe AS (n = 15)	Post-AVR/TAVI (n = 5)	*P* value
Post-AVR/TAVI time (day)	—	—	2897 (350, >30 years)	
Age (years)	78.0 ± 10.0 (61–95)	72.8 ± 8.3 (61–88)	68.0 ± 12.5 (54–82)	0.07
Male	7 (25.9%)	6 (40%)	3 (60%)	0.32
BMI (kg/m^2^)	25.7 ± 3.47 (17.7–32.4)	25.6 ± 3.69 (18.8–32.9)	24.4 ± 3.29 (20.5–29)	0.768
**Cardiac risk factors**				
Hypertension	21 (77.8%)	12 (80%)	2 (40%)	0.22
Diabetes mellitus	14 (51.9%)	4 (26.7%)	2 (40%)	0.35
Hyperlipidemia	15 (55.6%)	9 (60%)	1 (20%)	0.34
Smoking	6 (22.2%)	55 (33.3%)	0 (0%)	0.31
**Other comorbidities**				
ESRD	3 (11.1%)	2 (13.3%)	0 (0%)	>0.99
COPD/asthma	0 (0%)	1 (6.7%)	1 (20%)	0.18
Post pacemaker implantation	0 (0%)	0 (0%)	2 (40%)	0.009*
RBBB	2 (7.4%)	0 (0%)	1 (20%)	0.2

Values are presented as Mean ± SD (range) or N (%) as appropriate [exc. post-AVR/TAVI time is presented as median (range)].

*AVR*, aortic valve replacement; *TAVI*, transcatheter aortic valve implantation; *BMI*, body mass index; *ESRD*, end-stage renal disease, estimated glomerular filtration rate (eGFR) < 15 ml/min/1.73 m^2^; *COPD*, chronic obstructive pulmonary disease; *RBBB*, right bundle branch block. *P value < 0.05.

Most of the post-AVR/TAVI group had normal or slightly dilated LV, fair left ventricular ejection fraction (LVEF) (i.e. LVEF ≥50%) and a trans-valvular pressure gradient >20 mmHg before the AV intervention. The severity of AS greatly improved after the intervention [AV mean pressure gradient (PG) = 7.09 ± 4.62]. The time interval between the index MPI and AV intervention ranged from 350 days to more than 30 years.

Comparing groups, the patients in the prior AVR/TAVI group were relatively younger. Except for the higher proportion of patients receiving pacemaker implantation in the prior AVR/TAVI group (*P = *0.009), there was no significant differences in sex, body mass index (BMI), cardiac risk factors, and other comorbidities including end-stage renal disease, chronic obstructive pulmonary disease/asthma, and right bundle branch block.

### Resting doppler echocardiography (UCG)

The echocardiographic variables are summarized in Table 2. The patients with moderate-to-severe AS had a significantly higher grade of AS than not only those with mild AS but prior AVR/TAVI group (*P* < 0.001). The mean LVEF measured in the patients with mild AS showed marginal statistical significance compared with the moderate-to-severe AS and prior AVR/TAVI groups (*P* = 0.058 and 0.054, respectively). The AVR/TAVI group had a significantly lower atrial filling (A) velocity than the mild AS group (*P* = 0.023). However, there were no significant differences in left atrial (LA) and LV dimensions and other diastolic parameters between groups (*P* > 0.05).

**Table 2:** Comparisons of resting Doppler echocardiography in the three groups.

	Mild AS (n = 27)	Moderate-to-severe AS (n = 15)	Prior AVR/TAVI (n = 5)	*P* value
LA (mm)	38.1 ± 4.45	39.9 ± 4.89 (n = 14)	42 ± 6.16	0.18
IVS (mm)	11.9 ± 1.97	12.5 ± 1.64	11.4 ± 2.3	0.43
PW (mm)	11.5 ± 1.95	12.3 ± 1.5	11.8 ± 2.39	0.41
LVEDD (mm)	45.2 ± 6.68	47.5 ± 7.54	44.6 ± 4.51	0.45
LVESD (mm)	27 ± 4.75	30.3 ± 6.23	29.4 ± 4.78	0.18
LVEF_UCG_ (%)	70.2 ± 7.46	65 ± 9.3	62.2 ± 9.55	0.055^$^
AV peak flow (cm/s)	240 ± 42.3	324 ± 63.1	181 ± 61.9	0.001^#^
AV mean PG (mmHg)	11.6 ± 3.67	22.5 ± 8.62	7.09 ± 4.62	0.001^#^
TRPG (mmHg)	25.6 ± 13 (n = 14)	31.2 ± 15.1 (n = 9)	17.3 ± 1.89 (n = 4)	0.21
E (cm/s)	88.5 ± 27.7 (n = 24)	89.1 ± 37.6 (n = 14)	99.4 ± 52.9	0.99
A (cm/s)	120 ± 19.2 (n = 26)	111 ± 19.9 (n = 13)	89.8 ± 26.8 (n = 4)	0.03^*^
E/A ratio	0.73 ± 0.19 (n = 26)	0.73 ± 0.18 (n = 13)	1.02 ± 0.7 (n = 4)	0.14
DT (ms)	241 ± 96.2 (n = 25)	241 ± 153 (n = 14)	287 ± 150 (n = 4)	0.29

Values are presented as mean ± SD as appropriate.

*IVS*, interventricular septum; *PW*, posterior wall; *EDD*, end-diastolic dimension; *ESD*, end-systolic dimension; *PG*, pressure gradient; *TR*, tricuspid regurgitation; *E*, the peak early filling velocity of transmitral inflow; *A*, the late diastolic filling velocity of transmitral inflow; *DT*, deceleration time.

^$^LVEF_UCG_: *P* = 0.058 for mild AS vs. moderate-to-severe AS, *P* = 0.054 for mild AS vs. prior AVR/TAVI.

^#^AV peak flow and AV mean PG: *P* < 0.001 for mild AS vs moderate-to-severe AS, *P* < 0.001 for moderate-to-severe AS vs. prior AVR/TAVI.

*A: *P* = 0.023 for mild AS vs. prior AVR/TAVI.

### MPI parameters

**Figure 1:**
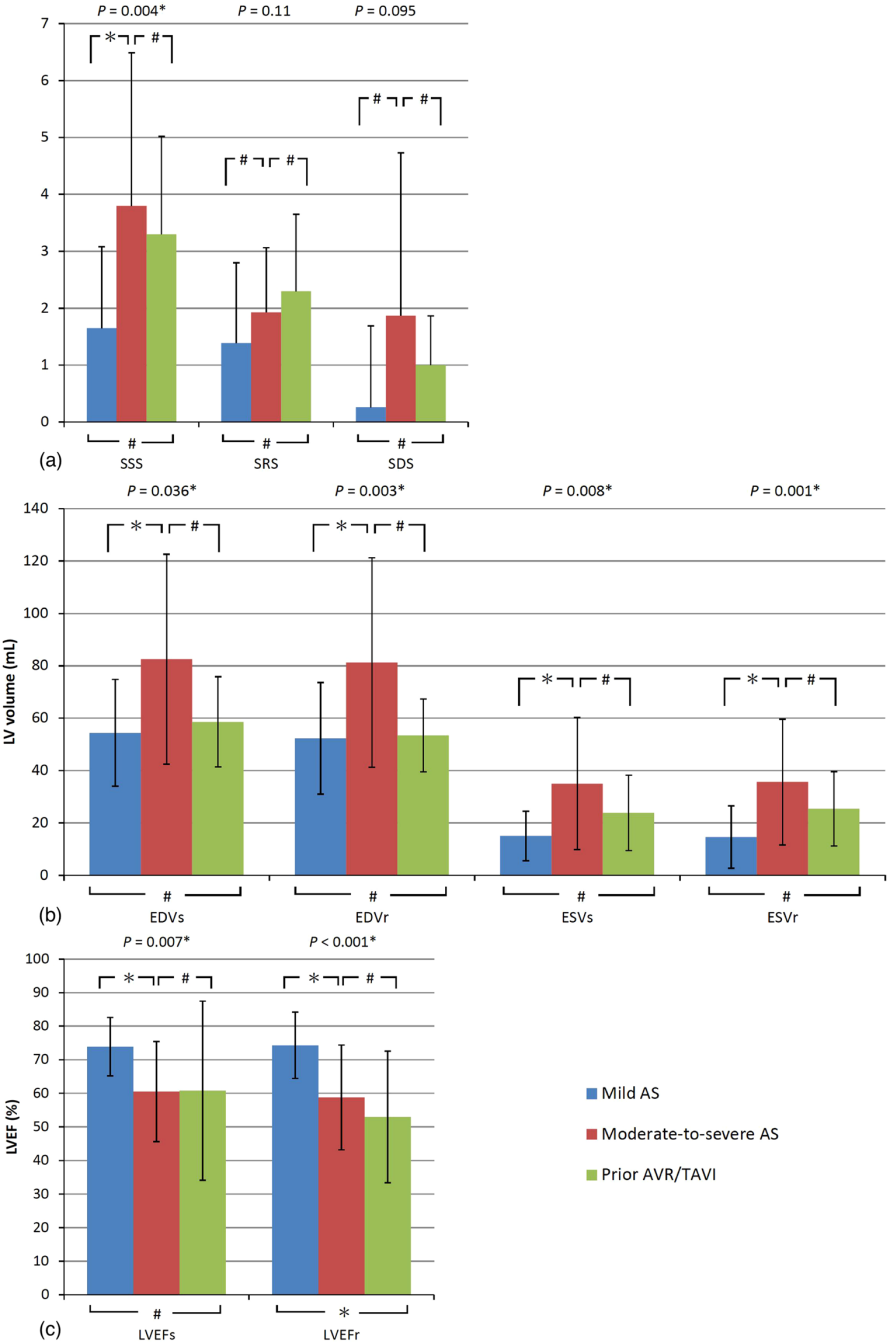
Comparison of the perfusion defects (**a**), LV volume (**b**) and LVEF (**c**) in three groups measured by SPECT MPI. Values are presented as mean ± SD or N (%) as appropriate. *SSS*, summed stress score; *SRS*, summed rest score; *SDS*, summed difference score; *LHRs or r*, lung/heart ratio after stress or at rest; *EDV*, end-diastolic volume; *ESV*, end-systolic volume; *LVEF*, left ventricular ejection fraction. *P value < 0.05; ^#^*P* value ≥ 0.05.

The MPI parameters are summarized in Table 3 and Fig. 1. The patients with moderate-to-severe AS had a larger LV size than the other groups, and the patients with moderate-to-severe AS and prior AVR/TAVI had a lower LVEF. Two patients (13.3%) in the moderate-to-severe AS group had marked RV uptake after stress. Summed stress score (SSS) was significantly higher in the moderate-to-severe AS group compared to the mild AS group (*P* = 0.007), but similar result between the moderate-to-severe AS and prior AVR/TAVI groups. There were no significant differences in summed rest score (SRS), summed difference score (SDS), post-stress or resting lung-heart ratio (LHRs or LHRr) or stress-induced increased LVEF (ΔLVEF = post-stress LVEF- resting LVEF) among the groups.

**Table 3:** Comparison of MPI results in the three groups.

	Mild AS (n = 27)	Moderate-to-severe AS (n = 15)	Prior AVR/TAVI (n = 5)	*P* value
SSS ≥ 4	4 (14.8%)	7 (46.7%)	2 (40%)	0.07
LHRs	0.35 ± 0.05	0.37 ± 0.07	0.39 ± 0.06	0.2
LHRr	0.37 ± 0.05	0.38 ± 0.07	0.36 ± 0.08	0.74
Marked RV uptake after stress	0 (0%)	2 (13.3%)	0 (0%)	0.18

Values are presented as mean ± SD or N (%) as appropriate.

*SSS*, summed stress score; *LHRs* or *r*, lung/heart ratio after stress or at rest; *RV*, right ventricular.

### Analysis of the contribution of SSS, SRS and SDS

Overall and subgroup analysis were performed to evaluate the contributors to SSS (Table 4, Supplementary Table S1). Increased AV PG, LHRs and LV volumetric data [including post-stress end-diastolic volume (EDVs) and end-systolic volume (ESVs), resting end-diastolic volume (EDVr) and end-systolic volume (ESVr)] were associated with a higher SSS. Due to obvious collinearity, EDVs was chosen as the representative of the LV volume parameters in the multivariate regression analysis. After the multivariate analysis, only increased LHRs and EDVs were independent contributors of SSS.

**Table 4:** Univariate and multivariate analyses of SSS contributors in logistic regression.

Characteristic	Univariate analysis				Multivariate analysis			
	OR	95% CI		*P* value	OR	95% CI		*P* value
		Lower	Upper			Lower	Upper	
Age	0.98	0.92	1.04	0.45	—	—	—	—
Male	0.31	0.08	1.17	0.08	—	—	—	—
Hypertension	1.44	0.35	5.97	0.61	—	—	—	—
Diabetes mellitus	0.53	0.15	1.93	0.34	—	—	—	—
Hyperlipidemia	0.4	0.1	1.53	0.18	—	—	—	—
Smoking	1.03	0.23	4.66	0.97	—	—	—	—
ESRD	1.6	0.16	15.8	0.69	—	—	—	—
LVEDD	1.09	0.99	1.2	0.08	—	—	—	—
LVESD	1.12	0.99	1.27	0.06	—	—	—	—
LVEF_UCG_	0.97	0.9	1.04	0.35	—	—	—	—
AV mean PG	1.09	1	1.19	0.047*	1	0.9	1.12	0.97
TRPG	1.02	0.96	1.09	0.46	—	—	—	—
LHRs	1.2	1.05	1.38	0.008*	1.2	1.03	1.39	0.02*
LHRr	0.99	0.89	1.1	0.82	—	—	—	—
Marked RV uptake	1.18	0.94	1.49	0.99	—	—	—	—
EDVs	1.07	1.02	1.12	0.003*	1.1	1.02	1.13	0.01*
ESVs	1.07	1.02	1.12	0.008*	—	—	—	—
LVEFs	0.98	0.94	1.02	0.27	—	—	—	—
EDVr	1.06	1.01	1.11	0.02*	—	—	—	—
ESVr	1.06	1.01	1.1	0.008*	—	—	—	—
LVEFr	0.97	0.93	1.01	0.13	—	—	—	—

*SSS*, summed stress score; *LHRs or r*, lung/heart ratio after stress or at rest; *EDV*, end-diastolic volume; *ESV*, end-systolic volume; *LVEF*, left ventricular ejection fraction. Other *abbreviations* as Tables 1–3. *P < 0.05.

On the other hand, increased LHRs was the only predictor to SRS (*P* = 0.014), and marginal significance of higher AV PG in multivariate analysis (*P* = 0.052) (see Supplementary Table S2). Larger LV volume were highly correlated with greater SDS (*P* = 0.022) (see Supplementary Table S3). There were no significantly different in patients with native or after AV interventions (see Supplementary Table S4).

In summary, AS severity and LHRs were correlated with higher SSS and SRS, but not SDS. LV volume parameters were independent predictors of SSS and SDS.

There was some difference in LVEF as measured by UCG and SPECT (Table 2 and Fig. 1c) (LVEF_UCG_ vs. LVEFr between each group, *P* = 0.055 vs. P < 0.001). Bland-Altman plot showed fair agreement but a regression line with a positive slope, indicating systemic bias existed (see Supplementary Fig. S1). Patients with small LV cavity (ESVs < 20 ml) on gated SPECT had more discrepancy of LVEF between UCG and SPECT (LVEFr-LVEF_UCG_ of ESVs < 20 ml vs. ESVs ≥20 ml, 6.1 ± 9.8 vs. −10.5 ± 7.4, *P* < 0.001), and higher prevalence of small hearts (ESVs < 20 ml) in mild AS group.

### Safety concerns of dipyridamole stress

Regarding the safety, dipyridamole induced adverse effects in the medical records from the initial study cohort (n = 102) were recorded (see Supplementary Table S5), and no severe adverse effects or emergent events were noted, even in patients with documented obstructive CAD (n = 36) and significant arrhythmia (n = 9).

## Discussion

### MPI presentation in patients with AS

Few studies have focused on the presentation of perfusion defects on MPI in patients with AS. Pfisterer *et al*. described reversible apical perfusion defects in patients with AS without CAD, and concluded that they were therefore not specific^16^. Rask *et al*. demonstrated normal thallium distribution in patients with AS, which tended to be lower in some regions than in patients without significant CAD^18,19^.

Dipyridamole induced perfusion defects was not uncommon in our overall AS cohort, although mostly mild, and SSS was positively correlated with degree of AS. An increasing number of studies have investigated MBF and MFR obtained from dynamic imaging protocols in recent years. Rajappen *et al*. reported that the decrease in MFR was correlated with the severity of AS and increased hemodynamic load, but not left ventricular hypertrophy (LVH)^4^. Furthermore, in whom post-AVR, the increase in hyperemic MBF and MFR was directly related to the increase in AV area^20^. Burwash *et al*. found that low-flow, low-gradient AS was characterized by higher resting MBF and reduced MFR, which were related to the severity of AS^6^. Carpeggiani *et al*. also found depressed MBF and MFR in severe AS patients, yet they were independent of LVH and AV PG^15^. No improvement of myocardial perfusion was noted despite the decrease in LV mass after interventions. We also noted similar phenomenon.

In the current study, we demonstrate that increased LHRs and LV volumes contributed to dipyridamole-induced perfusion abnormalities. LV size reflects the LV remodeling process. An increased lung to heart ratio (LHR) on stress MPI is a predictor of adverse cardiac events and identifies people with extensive CAD. It has been demonstrated to reflect stress-induced LV dysfunction in previous studies^21,22^.

### LV diastolic function in patients with aortic stenosis

The peak velocities of transmitral flow at early filling (E) to A (E/A ratio) reversal was noted in majority of patients with native valves, but not significantly correlated to the presence of LVH or systolic dysfunction (LVEDD and LVEF_UCG_, *P* > 0.05). Diastolic dysfunction starts before systolic dysfunction in patients with aortic valve disease^23,24^. In our study, most patients with native valves were in the earlier stages of AS, although with preserved LVEF, diastolic dysfunction are rather common.

### Difference in LVEF measurements by UCG and SPECT

Quantification of LVEF and LV volumes using gated SPECT was a reliable complementary tool^25,26^. Several factors may affect the accuracy, including LV size^27–29^, gender^30–32^, perfusion defects^33,34^ and technological issues. ^201^Tl is known to be a suboptimal radiopharmaceutical for gated SPECT because of low myocardial count densities and lower image resolution may create problems, especially in small hearts, in delineating the LV cavity^35^. Higher prevalence of small hearts (ESVs < 20 ml) in mild AS group.

LVEF by 2-dimensional echocardiography with modified Simpson’s biplane measurement were not routinely performed. LVEF and volumetric data derived by M-mode echocardiography are less accurate because by M-mode echocardiography has limitations, including 1) the mathematic and geometric assumptions used to derive the volumes from which the ejection fraction is calculated, and 2) the lack of applicability to asynergic ventricles^36^. These might be explained by LVEF differences between two methods.

### Indications and contraindications for MPI in Patients with aortic stenosis

Although the American College of Cardiology/American Heart Association Guidelines recommend that patients with AS having equivocal symptoms should receive modified exercise tests^5^, the role of vasodilator stress agents in these patients is controversial. In the current study, none of the AS patients experienced severe adverse events with unstable hemodynamics, which is similar to prior studies^10,12–14,18,37,38^. Scarsini *et al*. revealed substantial agreement between stress MPI and fractional flow reserve (FFR) and high negative predictive value (NPV) in identifying coronary lesions^39^. Our findings agreed that vasodilator stress can be a valuable tool to evaluate dipyridamole-induced ischemia in patients with AS and guide the treatment strategy.

### Limitations

This study was a retrospective study with a relatively small number of patients conducted at a single tertiary medical center, and therefore referral bias exists. The majority of patients had mild and moderate AS. We excluded patients with known significant CAD and impaired LV systolic function, thus the severity of ischemia and dipyridamole related adverse effects could be less severe than previous reports. Due to the retrospective nature, not all patients had coronary angiography within 6 months, and LVEF by two-dimensional echocardiography with modified Simpson’s biplane measurement were not routinely performed. Besides the small sample size, the interval between AV intervention and MPI was heterogeneous.

Further studies are merit to investigate the use of dipyridamole MPI in patients with severe AS, pre and post AV intervention, with a larger population and longer follow-up.

## Methods

### Study population and study design

**Figure 2:**
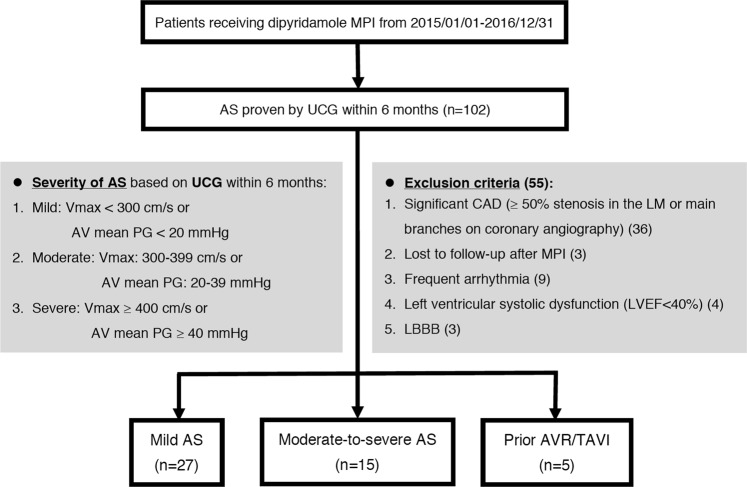
The inclusion algorithm of the study population. A total of 47 patients with both CZT-based MPI and Doppler echocardiography were included. *MPI*, myocardial perfusion imaging; *UCG*, echocardiography; *CAD*, coronary artery disease; *LM*, left main; *LVEF*, left ventricular ejection fraction; *LBBB*, left bundle branch block.

This study was approved by the Institutional Review Board of Far Eastern Memorial Hospital (107167-E). The need for written informed consent was waived due to the retrospective nature of the study. All procedures and methods were performed in accordance with the updated guidelines and regulations. We retrospectively analyzed data from the database of a medical center in Taiwan. We reviewed all patients who underwent ECG-gated dipyridamole MPI using a cadmium-zinc-telluride camera between 2015 and 2016, and those with valvular AS diagnosed by UCG and preserved LV function within 6 months were included. The inclusion algorithm of the study population is shown in Fig. 2. Patients with known CAD (those with coronary stenosis ≥50% in left main or three main branches, or who received previous PCI/coronary artery bypass surgery (CABG) without evidence of patent stents or grafts in the last 6 months), LVEF_UCG_ <40%, or loss of follow-up after MPI were excluded. To reduce the interference to ECG-gated SPECT imaging analysis, those with left bundle branch block (LBBB) or frequent arrhythmia, which meant frequent atrial or ventricular premature contractions (APCs or VPCs) or atrial fibrillation (Af) were also kept out of the study. The demographic data included age, sex, cardiac risk factors (hypertension, diabetes mellitus, hyperlipidemia, and smoking), and other comorbidities (end-stage renal disease, chronic obstructive pulmonary disease or asthma, post-pacemaker implantation, and right bundle branch block). Based on the AV mean PG and AV peak velocity (V_max_) recorded on UCG in the last 6 months, the population was divided into three groups: (1) mild AS (AV V_max_ < 300 cm/s or AV mean PG < 20 mmHg), (2) moderate-to-severe AS (AV V_max_ ≥ 300 cm/s or AV mean PG ≥ 20 mmHg), and (3) with prior AVR or TAVI. The clinical characteristics, safety of dipyridamole stress and MPI parameters were compared between the groups.

### Resting echocardiography

Resting echocardiography was performed by experienced cardiologists. M-mode and two-dimensional Doppler imaging data were collected. LA and LV dimensions and related indexes were measured. Diastolic parameters including E and A waves, derived E/A ratio, deceleration time (DT) at early filling and tricuspid regurgitation pressure gradient (TRPG) were recorded.

### Stress protocols and electrocardiogram-gated SPECT MPI

All patients were stable at the time of stress imaging, and they all received dipyridamole infusion over 4 minutes at a dose of 0.56 mg/kg. A dose of 2 mCi (74 MBq) thallium-201 was then injected at the 7th minute. The injected dose was 2.5 mCi (92.5 MBq) if the patient weighed more than 90 kg, and 3.0 mCi (111MBq) if they weighed more than 100 kg. At the 10th minute, routine 75–125 mg of aminophylline was injected intravenously for dipyridamole-induced adverse effects. Imaging began within 5 minutes after the aminophylline injection and was repeated 4 hours later. ECG, oxygen saturation and blood pressure were continuously monitored during the stress test. The development of any arrhythmia, ST segment changes, significant blood pressure (BP) changes or symptoms were recorded. ECG-gated SPECT MPI was performed in the supine position using a cadmium-zinc-telluride gamma camera (Discovery NM530c, GE Healthcare, Chicago, Illinois, USA) which was equipped with 19 pinhole collimators and 19 solid-state cadmium-zinc-telluride detectors along a 180-degree arc. Each detector was 8 × 8 cm in size, and projections were obtained using a 32 × 32 matrix (2.46 × 2.46 mm in size). The energy window was set as default for thallium-201: asymmetrically (−14% to +23%) at 70 keV, and symmetrically (−9% to +9%) at 167 keV. ECG-gating was also implemented using a built-in system^40–42^. Images were reconstructed with a maximum likelihood expectation maximization-based iterative algorithm (30 iterations for gated images, 70 iterations for non-gated images) using a Butterworth filter (order 15, cut-off frequency 0.28)^43,44^.

### Imaging analysis

SPECT quantitative perfusion and quantitative gated SPECT (QPS/QGS) software (Cedars-Sinai Medical Center, Los Angeles, CA) was used to analyze the gated images. Perfusion defects were read using a 17-segment model; segmental uptake was assessed using a 5-point scoring system (from 0 = normal uptake to 4 = absent uptake). SSS, SRS and SDS were derived. In addition, LHR, LVEF, volumetric and right ventricular (RV) uptake data were also calculated.

### Safety

Aminophylline injection was given in routine to resolve the side effect or to prevent late onset adverse effect during scanning. The safety of dipyridamole stress was assessed according to hemodynamic response, significant changes in ECG, stress-induced attacks of asthma or chronic obstructive pulmonary disease, and any adverse events recorded during the stress and rest tests. A significant hemodynamic response was defined as hypotension (BP <90 mmHg) and a drop in systolic blood pressure >40 mmHg after stress. Dipyridamole-induced arrhythmia and ST-T changes such as premature contraction, atrioventricular block, ST depression and T wave inversion were recorded. Chest pain and chest tightness were classified as chest discomfort. Dizziness, head fullness, headache or heavy headiness were classified as head discomfort. Other categories of side effects included abdominal discomfort, dyspnea, body soreness and hot flushes.

### Statistical analysis

Continuous variables are reported as mean ± standard deviation and categorical data as percentages, apart from post-AVR/TAVI time which is presented as median (range). Differences in continuous variables among groups were compared using analysis of variance (ANOVA) or the Kruskal-Wallis test, based on normal or non-normal data. The chi-square test was used for categorical data. Power transformation was performed to transform non-normal variables into normal variables. To determine the contributions of SSS, SRS and SDS, linear correlation and the logistic regression analyses focusing on SSS were performed. Besides the impact of AV severity, multivariate regression analysis was carried out to adjust for other covariates, using variables with *P* < 0.05 in univariate analysis. For parameters in collinearity, we chose one representative factor to undergo multivariate analysis. A *P* value < 0.05 was considered to be statistically significant. To assess relationships between AS severity and perfusion defects, the parameter AV mean PG was further separated by cutoff points of 20 mmHg. In addition, SSS data were also classified as being normal (SSS < 4) and abnormal (SSS ≥4), and logistic regression was used to analyze the contributors. Bland-Altman plot was further performed to compare the difference between LVEFr and LVEF_UCG_. SPSS 22 (IBM, USA) was used for the statistical analysis.

## Conclusions

Dipyridamole stress is clinically feasible with a reasonable degree of safety in patients with mild and moderate AS. Dipyridamole-induced perfusion abnormalities in patients with AS are not uncommon, but are mostly mild and in correlated to the AS severity. Valvular AS may be a potential cause of microvascular dysfunction. A normal MPI in a patient with documented AS could help clinicians to exclude the presence of obstructive CAD.

### New knowledge gained

Dipyridamole stress MPI could be safe in patients with mild and moderate AS. Normal MPI results can be helpful to exclude the possibility of CAD.

## Supplementary information

Supplementary Information

## Data Availability

The data are available from the corresponding author on reasonable request.
