# Supplementary material for: The clinical value and safety of ECG-gated dipyridamole myocardial perfusion imaging in patients with aortic stenosis
Source: Sci Rep. 2019 Aug 27;9:12443. doi: 10.1038/s41598-019-48901-y (PMC6712027; doi:10.1038/s41598-019-48901-y)

The clinical value and safety of ECG-gated dipyridamole myocardial perfusion imaging in patients with aortic stenosis

Fang-Shin Liu, MD, Shan-Ying Wang, MD, Yu-Chien Shiau, MD, Yen-Wen Wu, MD, PhD*

Supplementary Table S1. Univariate analyses of summed stress score (SSS) contributors showed male sex, elevated AV PG, increased LHRs and increased LV volume (including EDVs, ESVs, EDVr and ESVr) were in positive correlation, which remained significant in multivariate analysis, except elevated AV PG.

Characteristic	Univariate analysis			Multivariate analysis		
	Nonstandardization coefficient (SE)	Standardization coefficient	*P* value	Nonstandardization coefficient (SE)	Standardization coefficient	*P* value
Age	-0.04 (0.03)	-0.22	0.14	-	-	-
Male	1.92 (0.54)	0.47	0.001*	1.05 (0.48)	0.26	0.03*
Hypertension	-0.59 (0.66)	-0.13	0.38	-	-	-
DM	0.09 (0.59)	0.03	0.87	-	-	-
Hyperlipidemia	0.95 (0.57)	0.24	0.1	-	-	-
Smoking	0.38 (0.68)	0.08	0.59	-	-	-
ESRD	-0.82 (0.93)	-0.13	0.39	-	-	-
LVEDD	0.03 (0.04)	0.1	0.5	-	-	-
LVESD	0.07 (0.05)	0.18	0.23	-	-	-
$LVEF_{UCG}$	-0.04 (0.03)	-0.17	0.25	-	-	-
AV mean PG	0.06 (0.04)	0.24	0.11	-	-	-
Mild vs. significant AS	1.5 (0.7)	0.31	0.04*	0.42 (0.57)	0.09	0.47
TRPG	-0.003 (0.03)	-0.02	0.94	-	-	-
LHRs	15.6 (4.09)	0.5	<0.001*	10.6 (3.58)	0.34	0.005*
LHRr	0.14 (4.75)	0.004	0.98	-	-	-
Marked RV uptake	1.51 (1.99)	0.11	0.45	-	-	-
EDVs	0.04 (0.008)	0.54	<0.001*	0.02 (0.008)	0.37	0.003*
ESVs	0.05 (0.01)	0.45	0.002*	-	-	-
LVEFs	-0.02 (0.02)	-0.18	0.23	-	-	-
EDVr	0.03 (0.008)	0.49	<0.001*	-	-	-
ESVr	0.04 (.014)	0.41	0.005*	-	-	-
LVEFr	-0.03 (0.02)	-0.23	0.13	-	-	-

DM, diabetes mellitus; *ESRD*, end-stage renal disease, estimated glomerular filtration rate (eGFR) < 15 ml/min/1.73 m^2; *UCG*, echocardiography; *EDD*, end-diastolic dimension; *ESD*, end-systolic dimension; *LVEF*, left ventricular ejection fraction; *AV, aortic valve: PG*, pressure gradient; Mild vs. significant AS, AV mean PG subdivided as < 20 and ≥ 20 mmHg; *TR*, tricuspid regurgitation; *LHRs or r*, lung/heart ratio after stress or at rest; *EDV*, end-diastolic volume; *ESV*, end-systolic volume; *RV*, right ventricular. *$P < 0.05$.

Supplementary Table S2. Univariate analyses of summed rest score (SRS) contributors showed male sex, elevated AV PG and increased LHRs was associated with SRS. Increased LHRs was a predictor, and a positive tendency between AV PG and SRS presented in subsequent multivariate analysis.

Characteristic	Univariate analysis			Multivariate analysis		
	Nonstandardization coefficient (SE)	Standardization coefficient	*P* value	Nonstandardization coefficient (SE)	Standardization coefficient	*P* value
Age	0.001 (0.02)	0.01	0.94	-	-	-
Male	0.98 (0.35)	0.39	0.007*	0.63 (0.33)	0.25	0.06
Hypertension	-0.69 (0.4)	-0.25	0.09	-	-	-
DM	0.09 (0.37)	0.04	0.81	-	-	-
Hyperlipidemia	0.17 (0.36)	0.07	0.62	-	-	-
Smoking	-0.16 (0.42)	-0.06	0.71	-	-	-
ESRD	-0.19 (0.58)	-0.05	0.74	-	-	-
LVEDD	-0.01 (0.03)	-0.07	0.67	-	-	-
LVESD	0.02 (0.03)	0.07	0.63	-	-	-
$LVEF_{UCG}$	-0.03 (0.02)	-0.21	0.17	-	-	-
AV mean PG	0.04 (0.02)	0.25	0.1	-	-	-
Mild vs. significant AS	1.03 (0.41)	0.34	0.02*	0.74 (0.37)	0.26	0.05
TRPG	0.002 (0.02)	0.03	0.89	-	-	-
LHRs	8.73 (2.59)	0.45	0.002*	6.51 (2.54)	0.34	0.01*
LHRr	1.91 (2.91)	0.1	0.51	-	-	-
Marked RV uptake	-0.54 (0.88)	-0.09	0.54	-	-	-
EDVs	0.008 (0.006)	0.21	0.17	-	-	-
ESVs	0.009 (0.01)	0.14	0.35	-	-	-
LVEFs	0.003 (0.01)	0.04	0.79	-	-	-
EDVr	0.007 (0.006)	0.18	0.23	-	-	-
ESVr	0.008 (0.009)	0.12	0.43	-	-	-
LVEFr	-0.004 (0.01)	-0.05	0.77	-	-	-

Abbreviations as Supplementary Table S1. *$P < 0.05$.

Supplementary Table S3. Univariate analyses of summed difference score (SDS) contributors showed younger age, increased LV volume and decreased LVEF contributed to a higher SDS. Only a larger LV volume may have been a predictor of greater SDS based on multivariate linear regression analysis.

	Univariate analysis			Multivariate analysis		
**Characteristic**	**Nonstandardization coefficient (SE)**	**Standardization coefficient**	***P* value**	**Nonstandardization coefficient (SE)**	**Standardization coefficient**	***P* value**
Age	-0.07 (0.03)	-0.36	0.02*	-0.03 (0.03)	-0.18	0.22
Male	1.08 (0.58)	0.27	0.07	-	-	-
Hypertension	0.07 (0.65)	0.02	0.92	-	-	-
DM	-0.27 (0.58)	-0.07	0.65	-	-	-
Hyperlipidemia	1.02 (0.55)	0.27	0.07	-	-	-
Smoking	0.56 (0.66)	0.13	0.4	-	-	-
ESRD	-0.37 (0.92)	-0.06	0.69	-	-	-
LVEDD	0.05 (0.04)	0.17	0.26	-	-	-
LVESD	0.04 (0.05)	0.11	0.45	-	-	-
$LVEF_{UCG}$	0.005 (0.03)	0.02	0.89	-	-	-
AV mean PG	0.03 (0.04)	0.12	0.45	-	-	-
Mild vs. significant AS	0.53 (0.72)	0.11	0.46	-	-	-
TRPG	0.006 (0.03)	0.04	0.85	-	-	-
LHRs	4.62 (4.55)	0.15	0.32	-	-	-
LHRr	-2.66 (4.62)	-0.09	0.57	-	-	-
Marked RV uptake	2.63 (1.92)	0.2	0.18	-	-	-
EDVs	0.03 (0.008)	0.5	< 0.001*	0.02 (0.01)	0.39	0.02*
ESVs	0.05 (0.01)	0.48	0.001*	-	-	-
LVEFs	-0.04 (0.02)	-0.29	0.048*	-0.01 (0.02)	-0.07	0.62
EDVr	0.03 (0.008)	0.49	0.001*	-	-	-
ESVr	0.05 (0.01)	0.48	0.001*	-	-	-
LVEFr	-0.04 (0.02)	-0.32	0.03*	-	-	-

Abbreviations as Supplementary Table S1. *$P < 0.05$.

Supplementary Table S4-1. SSS contributor analysis between native valves with mild and moderate-to-severe AS. Multivariate analysis revealed that increased LHRs and increased LV volumes contributed to a higher SSS, but male sex, AV PG and LVEF were not as significant as analyzed with univariate regression.

	Univariate analysis			Multivariate analysis		
**Characteristic**	**Nonstandardization coefficient (SE)**	**Standardization coefficient**	***P* value**	**Nonstandardization coefficient (SE)**	**Standardization coefficient**	***P* value**
Age	-0.04 (0.03)	-0.19	0.23	-	-	-
Male	1.71 (0.6)	0.42	0.007*	0.84 (0.56)	0.21	0.14
Hypertension	-0.28 (0.74)	-0.06	0.7	-	-	-
DM	-0.26 (0.62)	-0.07	0.67	-	-	-
Hyperlipidemia	1.1 (0.59)	0.29	0.07	-	-	-
Smoking	0.59 (0.68)	0.14	0.39	-	-	-
ESRD	-0.66 (0.93)	-0.11	0.48	-	-	-
LVEDD	0.05 (0.04)	0.18	0.26	-	-	-
LVESD	0.09 (0.05)	0.26	0.11	-	-	-
$LVEF_{UCG}$	-0.05 (0.04)	-0.2	0.21	-	-	-
AV mean PG	0.09 (0.04)	0.37	0.02*	0.005 (0.04)	0.02	0.89
Mild vs. significant AS	1.73 (0.68)	0.38	0.02*	-	-	-
TRPG	0.01 (0.03)	0.09	0.7	-	-	-
LHRs	13.6 (4.51)	0.44	0.005*	8.24 (3.96)	0.26	0.045*
LHRr	-1.86 (5.07)	-0.06	0.72	-	-	-
Marked RV uptake	1.66 (1.96)	0.14	0.4	-	-	-
EDVs	0.04 (0.008)	0.61	<0.001*	0.03 (0.01)	0.44	0.01*
ESVs	0.06 (0.01)	0.55	<0.001*	-	-	-
LVEFs	-0.05 (0.02)	-0.37	0.02*	-0.009 (0.02)	-0.06	0.68
EDVr	0.04 (0.008)	0.59	<0.001*	-	-	-
ESVr	0.05 (0.013)	0.51	0.001*	-	-	-
LVEFr	-0.04 (0.02)	-0.31	0.04*	-	-	-

Abbreviations as Supplementary Table S1. *$P < 0.05$.

Supplementary Table S4-2. SRS contributor analysis between native valves with mild and moderate-to-severe AS. Male sex, elevated AV PG and increased LHRs were positively correlated, yet AV PG was the only predictor in multivariate analysis.

	Univariate analysis			Multivariate analysis		
**Characteristic**	**Nonstandardization coefficient (SE)**	**Standardization coefficient**	***P* value**	**Nonstandardization coefficient (SE)**	**Standardization coefficient**	***P* value**
Age	0.001 (0.02)	0.008	0.96	-	-	-
Male	0.89 (0.38)	0.35	0.02*	0.55 (0.36)	0.22	0.14
Hypertension	-0.72 (0.44)	-0.25	0.11	-	-	-
DM	-0.15 (0.38)	-0.06	0.7	-	-	-
Hyperlipidemia	0.18 (0.38)	0.08	0.64	-	-	-
Smoking	-0.04 (0.43)	-0.02	0.92	-	-	-
ESRD	-0.09 (0.58)	-0.03	0.87	-	-	-
LVEDD	-0.002 (0.03)	-0.01	0.94	-	-	-
LVESD	0.02 (0.04)	0.1	0.53	-	-	-
$LVEF_{UCG}$	-0.03 (0.02)	-0.18	0.26	-	-	-
AV mean PG	0.06 (0.02)	0.41	0.008*	-	-	-
Mild vs. significant AS	1.19 (0.4)	0.43	0.005*	0.9 (0.39)	0.33	0.03*
TRPG	0.009 (0.02)	0.11	0.62	-	-	-
LHRs	7.40 (2.86)	0.38	.013*	4.91 (2.76)	0.25	0.08
LHRr	2.08 (3.12)	0.11	0.51	-	-	-
Marked RV uptake	-0.45 (0.87)	-0.08	0.61	-	-	-
EDVs	0.01 (0.006)	0.27	0.09	-	-	-
ESVs	0.01 (0.01)	0.21	0.2	-	-	-
LVEFs	-0.002 (0.02)	-0.03	0.87	-	-	-
EDVr	0.01 (0.006)	0.26	0.1	-	-	-
ESVr	0.01 (0.009)	0.17	0.28	-	-	-
LVEFr	-0.002 (0.01)	-0.03	0.87	-	-	-

Abbreviations as Supplementary Table S1. $^*P < 0.05$.

Supplementary Table S4-3. SDS contributor analysis between native valves with mild and moderate-to-severe AS. In univariate contributor analysis, A younger age, hyperlipidemia, larger LV size and decreased LVEF were correlated with a higher SDS. Only hyperlipidemia remained significant in multivariate analysis.

	**Univariate analysis**			**Multivariate analysis**		
**Characteristic**	**Nonstandardization coefficient (SE)**	**Standardization coefficient**	***P* value**	**Nonstandardization coefficient (SE)**	**Standardization coefficient**	***P* value**
Age	-0.07 (0.03)	-0.32	.039*	-0.02 (0.03)	-0.09	0.56
Male	1.05 (0.66)	0.25	0.12	-	-	-
Hypertension	0.40 (0.76)	0.08	0.61	-	-	-
DM	-0.36 (0.64)	-0.09	0.58	-	-	-
Hyperlipidemia	1.25 (0.6)	0.32	0.045*	1.16 (0.54)	0.29	0.04*
Smoking	0.66 (0.7)	0.15	0.35	-	-	-
ESRD	-0.31 (0.96)	-0.05	0.75	-	-	-
LVEDD	0.06 (0.04)	0.2	0.21	-	-	-
LVESD	0.06 (0.06)	0.16	0.33	-	-	-
$LVEF_{UCG}$	-0.003 (0.04)	-0.01	0.94	-	-	-
AV mean PG	0.04 (0.04)	0.14	0.38	-	-	-
Mild vs. significant AS	0.62 (0.75)	0.13	0.42	-	-	-
TRPG	0.02 (0.03)	0.11	0.64	-	-	-
LHRs	3.86 (5.12)	0.12	0.46	-	-	-
LHRr	-4.97 (5.17)	-0.15	0.34	-	-	-
Marked RV uptake	2.69 (2.00)	0.21	0.19	-	-	-
EDVs	0.03 (0.009)	0.5	0.001*	0.02 (0.01)	0.28	0.12
ESVs	0.05 (0.01)	0.52	0.001*			
LVEFs	-0.06 (0.02)	-0.42	0.006*	-0.04 (0.03)	-0.27	0.12
EDVr	0.03 (0.008)	0.52	0.001*	-	-	-
ESVr	0.05 (0.01)	0.53	<0.001*	-	-	-
LVEFr	-0.06 (0.02)	-0.42	0.007*	-	-	-

Abbreviations as Supplementary Table S1. *$P < 0.05$.

Supplementary Table S5. Safety concerns of MPI in initial AS population (n=102). No severe adverse effects or emergent events were noted after dipyridamole stress.

	Mild AS (n=42)	Moderate-to-severe AS (n=29)	Prior AVR/TAVI (n=31)	*P* value
**Significant BP change**				
SBP < 90 mmHg	4 (9.5%)	0 (%)	1 (3.2%)	0.18
SBP fall > 40mmHg	3 (7.1%)	0 (%)	1 (3.2%)	0.38
**Significant ECG change**				
Arrhythmia	1 (2.4%)	1 (3.4%)	2 (6.5%)	0.81
ST-T change	1 (2.4%)	1 (3.4%)	0 (%)	0.75
**Chest discomfort**				0.14
Mild	13 (31%)	10 (34.5%)	3 (9.7%)	
Severe	1 (2.4%)	0 (0%)	1 (3.2%)	
**Head discomfort**	9 (21.4%)	4 (13.8%)	8 (25.8%)	0.53
**Abdominal discomfort**	1 (2.4%)	3 (10.3%)	2 (6.5%)	0.37
**Dyspnea**	2 (4.8%)	2 (6.9%)	3 (9.7%)	0.88
**Body soreness**	1 (2.4%)	1 (3.4%)	3 (9.7%)	0.45
**Hot flush**	5 (11.9%)	5 (17.2%)	3 (9.7%)	0.7

Values are presented as N (%).

BP, blood pressure; *SBP*, systolic BP; *ECG*, electrocardiogram; *ST-T*, ST-T segment on electrocardiogram.

Supplementary Fig. S1: The graph created with Bland–Altman plot of resting LVEF measured with SPECT (LVEFr) in comparison with UCG ($LVEF_{UCG}$). It showed good agreement of LVEFr and $LVEF_{UCG}$ but the LVEF difference was significantly correlated with LV systolic function (r = 0.61, P < 0.0001). *LOA*, limits of agreement.

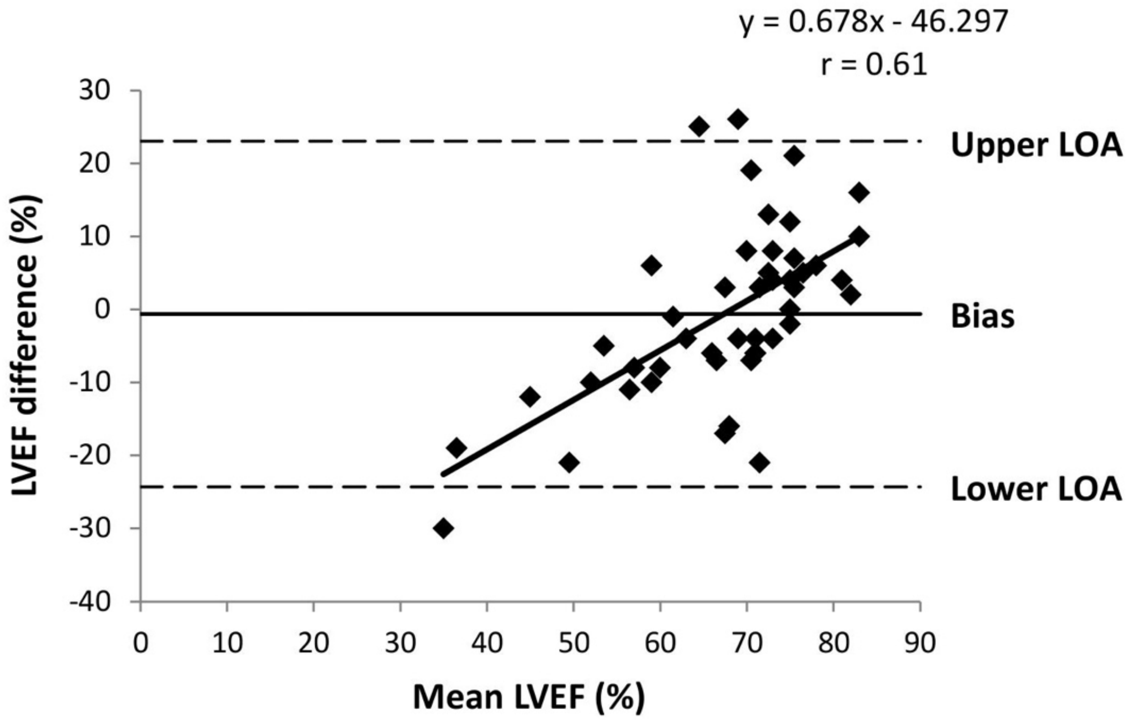

Supplement: Supplementary file 1 — Supplementary Information [file 41598_2019_48901_MOESM1_ESM.pdf]
